# Value of spectral detector computed tomography for the early assessment of technique efficacy after microwave ablation of hepatocellular carcinoma

**DOI:** 10.1371/journal.pone.0252678

**Published:** 2021-06-15

**Authors:** Robert Peter Reimer, Nils Große Hokamp, Julius Niehoff, David Zopfs, Simon Lennartz, Mariam Heidar, Roger Wahba, Dirk Stippel, David Maintz, Daniel Pinto dos Santos, Christian Wybranski

**Affiliations:** 1 Faculty of Medicine and University Hospital Cologne, Department of Diagnostic and Interventional Radiology, University of Cologne, Cologne, Germany; 2 Department of Radiology, Massachusetts General Hospital, Boston, MA, United States of America; 3 Faculty of Medicine, University Cologne, Cologne, Germany; 4 Faculty of Medicine and University Hospital Cologne, Department of General-, Visceral, Cancer and Transplant Surgery, University of Cologne, Cologne, Germany; Semmelweis University, HUNGARY

## Abstract

**Objectives:**

To investigate whether virtual monoenergetic images (VMI) and iodine maps derived from spectral detector computed tomography (SDCT) improve early assessment of technique efficacy in patients who underwent microwave ablation (MWA) for hepatocellular carcinoma (HCC) in liver cirrhosis.

**Methods:**

This retrospective study comprised 39 patients with 49 HCC lesions treated with MWA. Biphasic SDCT was performed 7.7±4.0 days after ablation. Conventional images (CI), VMI and IM were reconstructed. Signal- and contrast-to-noise ratio (SNR, CNR) in the ablation zone (AZ), hyperemic rim (HR) and liver parenchyma were calculated using regions-of-interest analysis and compared between CI and VMI between 40–100 keV. Iodine concentration and perfusion ratio of HR and residual tumor (RT) were measured. Two readers evaluated subjective contrast of AZ and HR, technique efficacy (complete vs. incomplete ablation) and diagnostic confidence at determining technique efficacy.

**Results:**

Attenuation of liver parenchyma, HR and RT, SNR of liver parenchyma and HR, CNR of AZ and HR were significantly higher in low-keV VMI compared to CI (all p<0.05). Iodine concentration and perfusion ratio differed significantly between HR and RT (all p<0.05; e.g. iodine concentration, 1.6±0.5 vs. 2.7±1.3 mg/ml). VMI_50keV_ improved subjective AZ-to-liver contrast, HR-to-liver contrast, visualization of AZ margin and vessels adjacent to AZ compared to CI (all p<0.05). Diagnostic accuracy for detection of incomplete ablation was slightly higher in VMI_50keV_ compared to CI (0.92 vs. 0.89), while diagnostic confidence was significantly higher in VMI_50keV_ (p<0.05).

**Conclusions:**

Spectral detector computed tomography derived low-keV virtual monoenergetic images and iodine maps provide superior early assessment of technique efficacy of MWA in HCC compared to CI.

## Introduction

Hepatocellular carcinoma (HCC) is a global health burden. It represents the fifth most common cancer type and the second most common cause of cancer-related death [1,2]. The incidence of HCC is continuously rising and will likely increase by >50% until the end of the decade [3].

Different treatment options for HCC are available depending on the tumor stage. For early, unresectable HCC, percutaneous or intraoperative thermal ablation is the standard therapy according to international management guidelines due to its efficacy, favourable safety profile, minimal invasiveness and potential to preserve hepatic parenchyma [1,4–6]. The European Association for the Study of Liver and the European Society of Medical Oncology even recommend thermal ablation as an alternative to surgical resection for Barcelona Clínic Liver Cancer stage 0 tumors [1,4,5]. During the last decade, microwave ablation (MWA) has emerged as a new thermal ablation technique. It applies rapidly oscillating electromagnetic fields at frequencies between 0.92–2.45 GHz. MWA agitates dipole water molecules that continuously realign along field orientation, inducing frictional heat and thus coagulative tissue necrosis [7–9]. MWA yields comparable results as the long-term established radiofrequency ablation (RFA) with regards to efficacy, long-term survival benefit and safety profile [10–12].

After MWA, cross-sectional follow-up imaging is necessary to monitor technique efficacy (completeness of ablation), local tumor progression, local tumor recurrence and distant tumor progression. [4,13]. Multiphasic contrast-enhanced computed tomography is currently the most used modality for follow-up due to its broad availability in liver cancer centres, robustness and high reproducibility [14–20]. In the past decade, dual-energy computed tomography systems (DECT) raised interest in the field of liver imaging due to its improved soft-tissue contrast and thus liver lesion conspicuity by means of low-keV virtual monoenergetic images (VMI) and its inherent feasibility to quantify iodine concentration [21–25]. Recently, a detector based DECT system has been introduced into clinical routine, referred to as spectral detector CT (SDCT). SDCT employs a single X-ray tube and separately registers low- and high-energy photons in two parallel stacked detector layers as opposed to DECT systems in which the dual-energy component is implemented at the level of the X-ray source. The independent registration of high- and low-energy attenuation characteristics enables reconstruction of VMI with energy levels between 40–200 keV and material specific iodine maps [26–28].

Several preclinical experimental studies evaluated the potential of DECT for early detection of residual tumor (RT) after thermal liver ablation. These studies evaluated its potential to directly quantify iodine concentration as a surrogate of perfusion by showing better correlation with technical success and differentiation of reactive hyperemic rim (HR) and RT [29,30]. However, clinical data of DECT for the early assessment of technique efficacy following thermal ablation is scarce [31–33]. Only one study analyzed the value of DECT in the immediate follow-up of HCC and liver metastases treated by RFA [34].

The aim of this study was to investigate whether the increased soft tissue contrast of VMI derived from contrast-enhanced SDCT along with iodine maps facilitate an improved early assessment of technique efficacy after MWA of HCC.

## Material and methods

### Study population

The local institutional review board approved this retrospective analysis (reference number: 18–226; Ethikkomission der Medizinischen Fakultät der Universität zu Köln) and waived the need for patient consent. All data were fully anonymized prior to the data analysis and all methods were performed in accordance with the relevant guidelines and regulations. Patients’ medical records within the local institutional (University of Cologne, University Hospital Cologne, Germany) radiology information system were accessed during June 2019 and June 2020. A structured query to the radiology information system was performed using the following inclusion criteria: 1) patients treated for HCC by MWA, 2) pre-treatment contrast-enhanced MRI or CT, 3) early post-treatment contrast-enhanced SDCT examination 5–21 days after MWA between May 2016 and January 2019 with a standardized imaging protocol. Imaging studies were reviewed using a clinical DICOM-Viewer (Impax EE R20, Dedalus Group). Hanging protocols indicating the HCC lesion and the ablation zone (AZ) were saved to guarantee reliable identification for quantitative and qualitative analysis.

### Ablation procedure

The decision for MWA treatment was derived in the institutional multidisciplinary liver tumor board for each case according to clinical guidelines. Ultra-sound guided MWA was performed during laparotomy (n = 8, 16.3%) or laparoscopy (n = 9, 18.4%) employing a 2.45-GHz clinical microwave ablation system (Solero Microwave Tissue Ablation System, AngioDynamics) by one of two liver surgeons (DS and RW) with 19 and 12 years of experience, respectively. CT-guided MWA (n = 32, 65.3%), employing a 2.45-GHz clinical microwave ablation system (AMICA-GEN Hybrid System, HS Hospital Service) was performed by one of two interventional radiologists (CW and DPS) with 7 and 5 years of experience in liver ablation, respectively. All procedures were performed under general anesthesia. Needle placement and energy application according to the manufacturer’s protocols aimed for an AZ safety margin of ≥ 5 mm in all directions around the tumor. Overlapping ablations for large lesions or suboptimal initial needle placement were performed if considered necessary. Needle tract ablation was applied according to the manufacturer’s recommendations.

### Image acquisition

All CT scans were performed for clinical indications on the same spectral detector CT scanner (IQon Spectral CT, Philips Healthcare). Patients were scanned in a head first, supine position. The institutional CT protocol for tumor evaluation following MWA comprises an arterial and a portal-venous phase image acquisition of the liver. Administration of 100 ml non-ionic, iodinated contrast media bolus (Accupaque 350 mg/ml, GE Healthcare) followed by a 30 ml saline chaser is routinely performed with an automated injection system at a flow rate of 3.5 ml/s (MEDRAD^®^ Stellant^®^, Bayer Vital AG). Bolus-tracking technique (threshold of 150 Hounsfield Units (HU) in the abdominal aorta) was used and image acquisition started with a delay of 15 and 50 s, respectively. Tube current modulation was activated in all patients (DoseRight 3D-DOM; Philips Healthcare). Detailed scanning parameters are reported in Table 1.

**Table 1:** Scan parameters.

Phase	Collimation (mm)	Pitch	Tube voltage (kVp)	Rotation Time (s)	Tube Current-Time Product (mAs)	CTDI_vol_ (mGy)
Arterial	64 x 0.625	0.485	120	0.5	150.3 ± 75.8	13.7 ± 6.8
Portal- venous				0.33	154.7 ± 74.2	14.0 ± 6.7

DLP, dose-length product; CTDI_vol_, volumetric CT dose index. Results are means ± standard deviation if appropriate.

### Image reconstruction

Conventional images (CI) were reconstructed using a hybrid iterative reconstruction algorithm (iDose^4^, Philips Healthcare) and a standard body soft tissue kernel (kernel B). VMI at energy levels of 40–100 keV with 10-keV increment were reconstructed with a dedicated spectral reconstruction algorithm (Spectral, Kernel B; Philips Healthcare). Denoising was set to a medium level (level 3 of 7). VMI at 50 keV (VMI_50keV_) were chosen as the preferred VMI reconstruction for qualitative image analysis for both, arterial and portal-venous phase images in a preliminary evaluation of 10 patients (not included in the study population) and in consideration of the known improvement of liver lesion conspicuity in low-keV VMI [22,23,25,35]. All images were reconstructed with a slice thickness of 2 mm and a section increment of 1 mm. Material specific iodine maps allowing for iodine concentration measurements (in mg/ml) in any given region of interest were generated [36].

### Follow-up evaluation and reference standard determination

Institution’s standard operation procedure included an early follow-up by SDCT one week after MWA as baseline, followed by contrast-enhanced CT and/or MRI scans at 3, 6, 9 and 12 months and every 6 months thereafter. Outcome (technique efficacy–completeness of ablation, local tumor progression, distant intrahepatic and extrahepatic tumor progression) was reported according to a standardized terminology [13]. Data was retrieved from imaging diagnostic reports and clinical records. To ensure an accurate ground truth regarding therapy outcome, images were retrospectively evaluated by one board-certified, attending radiologist (CW) with 8 years of experience in hepatic MWA to double-check the corresponding diagnoses and unequivocally determine the outcome in case of ambiguous reports.

### Quantitative analysis

Quantitative analysis was performed by region-of-interest-based measurements using a dedicated platform provided by the vendor (IntelliSpace Portal v.11; Philips Healthcare), which automatically quantified attenuation in HU and standard deviation (SD) of the CT numbers within the corresponding region of interest (ROI). All ROIs were placed on CI and copied to all reconstructed VMI and iodine maps to ensure consistency of the measurements. In arterial and portal-venous phase, two ROIs were placed in the AZ avoiding central charred tissue and two ROIs were placed in adjacent normal liver parenchyma. In arterial phase, two additional ROIs were placed within the HR around the AZ or in viable RT if present, as well as in the aorta to calculate perfusion ratios of HR and RT as previously reported [30,37,38]. All ROIs were ellipsoid (at least 0.5 cm^2^). Multiple measurements within the same structure were averaged. SD of the HU values of normal liver parenchyma (SD_Liver_) was considered a surrogate for image noise. Iodine concentration [mg/ml] of HR and RT was directly quantified by IntelliSpace Portal. Signal-to-noise ratio (SNR) of AZ, HR, RT and normal liver parenchyma (SNR_AZ_, SNR_HR_, SNR_RT_, SNR_Liver_) were calculated according to the following formula as previously reported [23,39,40]:

$$SNR=\frac{{HU}_{Structure}}{{SD}_{Structure}}$$ (1)

AZ-to-liver, HR-to-liver and RT-to-liver contrast-to-noise ratio (CNR) were defined as being adapted from previous [23,39,40]:

$${CNR}_{AZ}=|(HU_{liver}-{HU}_{AZ})|/{SD}_{liver}$$ (2)

$${CNR}_{HR}=|(HU_{liver}-{HU}_{HR})|/{SD}_{liver}$$ (3)

$${CNR}_{RT}=|(HU_{liver}-{HU}_{RT})|/{SD}_{liver}.$$ (4)

Perfusion ratios of HR and RT were calculated to normalize iodine concentrations and improve their reproducibility as previously reported [30,37,38]:

$$Perfusion\;ratio={Iodine\;concentration}_{ROI}/{Iodine\;concentration}_{Aorta}.$$ (5)

### Qualitative analysis

Anonymized and randomized early follow-up images were assessed independently by 2 radiologists (JN and RPR) with 4 and 3 years of experience respectively in follow-up imaging of hepatic MWA. Readers were blinded to clinical information and later follow-up exams.

In a first reading session, readers assessed multi-planar reconstructions (MPR) of the pre-interventional MRI or CT scans and early post-interventional arterial and portal-venous phase CI in a side-by-side comparison, according to routine clinical practice. The pre- and post-interventional image sets were co-registered by a semi-automatic algorithm provided by the DICOM viewer (Impax EE R20, Dedalus Group). The readers were allowed to adjust window settings, if needed.

At the second reading session, co-registered MPR of pre-interventional examinations and early post-interventional VMI_50keV_ of arterial and portal-venous scans were assessed in an analogous approach to the first reading session after a waiting period of 6 weeks to minimize a potential recall bias.

In the two reading sessions, both readers assessed overall shape of the AZ (spherical vs. irregular), conspicuity of AZ margin (diffuse, moderate, sharp) and manifestation of HR (visible, absent, discontinuous, completely surrounding AZ) according to the study by Schraml et al. [41]. AZ-to-liver contrast, HR-to-liver contrast, vessel depiction adjacent to AZ and diagnostic confidence in determination of technique efficacy were rated with 5-point Likert scales (Table 2) similar to Lee et al. [34]. Technique efficacy was rated as incomplete (AZ does not cover tumor completely) or complete (AZ covers tumor completely).

**Table 2:** Likert scales of qualitative analysis.

Likert scale	AZ-to-liver contrast	HR-to-liver contrast	Vessel depiction adjacent to AZ	Diagnostic confidence
1	none	none	none	none
2	low	low	low	low
3	moderate	moderate	moderate	moderate
4	high	high	high	high
5	extraordinary	extraordinary	extraordinary	extraordinary

AZ, ablation zone; HR, hyperemic rim.

### Statistical analysis

All analyses were performed using JMP Software (Version 14, SAS Institute) unless specified below. Non-parametric Steel Dwass test (all pairs) was performed to compare quantitative and qualitative results between CI and VMI. Wilcoxon test was used to compare iodine concentration and perfusion ratio between HR and RT. Sensitivity and specificity of the diagnosis incomplete ablation were calculated using a contingency table. Inter- and intra-rater reliability was determined by means of intraclass correlation coefficients (ICC) using R Studio (Version 1.1.456; RStudio) based on a mean of 2 raters, consistency, 2-way mixed-effects model for the qualitative analysis [42]. Inter-rater agreement was evaluated as described earlier: excellent (ICC > 0.8), good (ICC > 0.6), moderate (ICC > 0.4), and poor agreement (ICC < 0.4) [43]. A p-value < 0.05 was considered significant. Continuous variables are reported as mean ± SD and Likert scores as median (quartiles).

## Results

### Study cohort

The study cohort comprised 39 patients (mean age: 67.5 ± 8.8 years, male/female: 27/12), yielding a total of 49 HCC lesions treated with MWA. Mean largest HCC diameter was 1.9 ± 0.8 cm (range: 1 – 3.8 cm). Mean duration of MWA treatment was 9.0 ± 4.3 minutes (range: 3 – 21 minutes), mean applied energy was 45.2 ± 22.2 kJ (range: 14.4 – 126 kJ). SDCT was performed 7.7 ± 4.0 days (range: 5–15 days) after MWA. The mean follow-up period after MWA treatment was 431.7 ± 317.1 days (range: 108 – 1195 days). Treatment response is reported in Table 3.

**Table 3:** Treatment response to microwave ablation.

Treatment response	Patients, n (%)	Lesions, n (%)	Time since ablation, mean days ± SD (range)
No progression	19 (48.7)	22 (44.9)	467.8 ± 260.4 (193–1183)
Incomplete ablation	4 (10.3)	6 (12.2)	7.0 ± 1.4 (6–9)
Local tumor progression	2 (5.1)	2 (4.1)	116.0 ± 11.3 (108–124)
Intrahepatic progression	9 (23.1)	10 (20.4)	285.0 ± 161.4 (99–573)
Intra- and extrahepatic progression	2 (5.1)	4 (8.2)	58.0 ± 24.0 (41–75)
Extrahepatic progression	3 (7.7)	5 (10.2)	197.3 ± 186.7 (89–413)

n, number; SD, standard deviation.

### Quantitative analysis

Results comprise measurements of arterial and portal-venous phase, unless specified otherwise. Refer to Table 4 for detailed values.

**Table 4:** Results of quantitative analysis.

	CI	VMI_40keV_	VMI_50keV_	VMI_60keV_	VMI_70keV_	VMI_100keV_	VMI_200keV_
*Arterial Phase*							
Noise _SD-Liver_	17.6 ± 3.5	17.5 ± 4.8	15.3 ± 3.4	14.2 ± 3.0	13.6 ± 2.9	13.0 ± 2.9	12.8 ± 2.9
Attenuation _Liver_	68.6 ± 10.2	110.4 ± 36.4	89.2 ± 22.8	76.6 ± 14.9	69.2 ± 10.5	59.3 ± 5.7	53.6 ± 5.0
Attenuation _AZ_	44.2 ± 7.4	40.3 ± 14.2	42.2 ± 10.3	43.3 ± 8.3	43.9 ± 7.2	44.8 ± 6.2	45.3 ± 5.9
Attenuation _RT_	129.6 ± 24.8	308.5 ± 104.4	216.6 ± 65.0	162.6 ± 42.1	130.2 ± 28.3	87.5 ± 12.0	62.8 ± 8.1
Attenuation _HR_	90.9 ± 15.9	189.1 ± 46.3	138.9 ± 30.2	109.4 ± 21.1	91.9 ± 15.9	68.5 ± 9.8	55.0 ± 7.7
SNR _Liver_	4.1 ± 1.2	6.8 ± 3.2	6.2 ± 2.5	5.7 ± 2.1	5.4 ± 1.9	4.9 ± 1.6	4.5 ± 1.5
SNR _AZ_	2.7 ± 0.8	2.7 ± 1.4	3.1 ± 1.3	3.3 ± 1.2	3.4 ± 1.2	3.6 ± 1.2	3.7 ± 1.2
SNR _RT_	8.7 ± 6.2	19.1 ± 19.8	15.4 ± 13.7	12.9 ± 10.1	11.1 ± 7.8	8.4 ± 4.7	6.5 ± 3.2
SNR _HR_	5.3 ± 2.1	9.7 ± 4.2	8.4 ± 3.4	7.5 ± 2.9	6.7 ± 2.5	5.5 ± 1.8	4.6 ± 1.3
CNR _AZ_	1.4 ± 0.6	4.3 ± 2.3	3.2 ± 1.6	2.5 ± 1.2	1.9 ± 0.9	1.2 ± 0.6	0.7 ± 0.5
CNR _RT_	5.1 ± 3.0	17.7 ± 9.5	13.5 ± 6.8	10.2 ± 5.0	7.9 ± 3.9	4.1 ± 2.0	1.6 ± 1.1
CNR _HR_	1.3 ± 0.8	4.6 ± 2.5	3.3 ± 1.7	2.3 ± 1.2	1.7 ± 0.9	0.8 ± 0.5	0.5 ± 0.4
*Portal-venous Phase*							
Noise _SD-Liver_	17.4 ± 3.2	17.1 ± 4.0	15.1 ± 3.0	14.1 ± 2.7	13.5 ± 2.6	13.0 ± 2.6	12.8 ± 2.5
Attenuation _Liver_	103.0 ± 19.4	229.6 ± 65.7	165.6 ± 41.8	127.9 ± 28.0	105.5 ± 19.8	75.6 ± 9.9	58.4 ± 6.5
Attenuation _AZ_	45.9 ± 8.0	39.5 ± 16.3	42.2 ± 11.4	43.7 ± 8.8	44.7 ± 7.4	45.9 ± 5.9	46.6 ± 5.5
SNR _Liver_	6.2 ± 2.3	14.4 ± 6.6	11.7 ± 5.2	9.7 ± 4.1	8.3 ± 3.4	6.1 ± 2.1	4.8 ± 1.3
SNR _AZ_	2.7 ± 0.8	2.7 ± 1.3	3.0 ± 1.1	3.2 ± 1.0	3.4 ± 1.0	3.5 ± 0.9	3.6 ± 0.9
CNR _AZ_	3.5 ± 1.7	11.9 ± 5.9	8.8 ± 4.5	6.4 ± 3.4	4.8 ± 2.6	2.4 ± 1.3	1.0 ± 0.6

CI, conventional images; VMI, virtual monoenergetic images; SD, standard deviation; AZ, ablation zone; RT, residual tumor; HR, hyperemic rim; SNR, signal-to-noise ratio; CNR, contrast-to-noise ratio. Results are mean ± standard deviation.

#### Image noise

Image noise (SD_Liver_) was significantly lower in VMI_60-100keV_ of arterial phase images and in VMI_50-100keV_ in portal-venous phase images compared to CI, respectively (all p<0.05).

#### Attenuation

**Fig 1:**
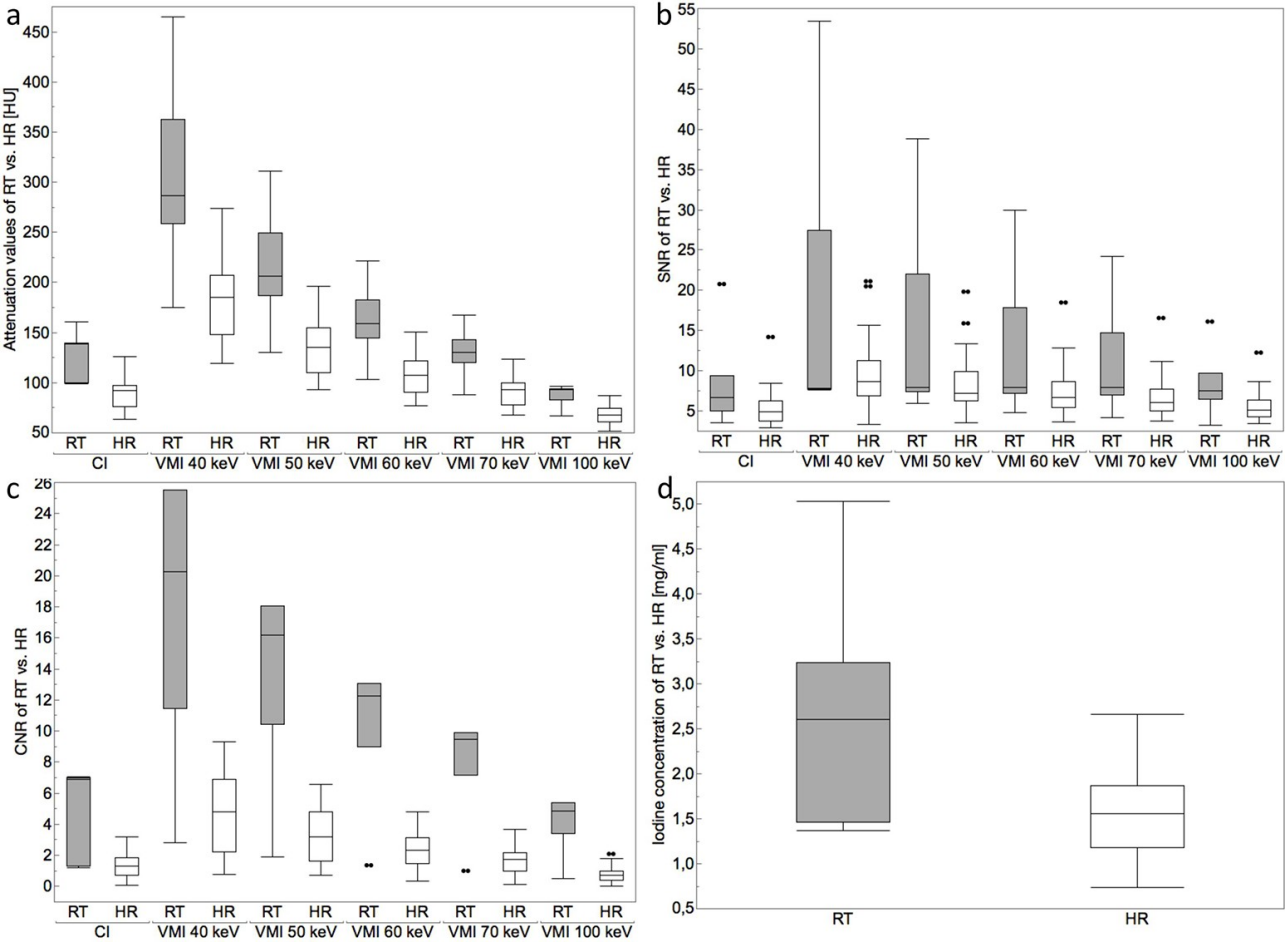
Illustrations of mean attenuation values (HU), signal-to-noise ratio (SNR), contrast-to-noise-ratio (CNR) and iodine concentration [mg/ml] reveal significantly higher values in residual tumor (RT) as compared hyperemic rim (HR) (a–d). Furthermore, attenuation and CNR were significantly higher in low-keV VMI compared to CI (a, c).

Attenuation of normal liver parenchyma in arterial phase VMI_40-50keV_ and in portal-venous phase VMI_40-60keV_ was significantly higher compared to CI (all p<0.05). Attenuation of HR in arterial phase VMI_40-60keV_ and of RT in arterial phase VMI_40keV_ was significantly higher compared to CI (both p<0.05). AZ attenuation remained constant in CI and in VMI at all keV levels (p≥0.05). RT showed significantly higher attenuation than HR in arterial phase images of respective image reconstructions (all p<0.05; Fig 1A).

#### Signal to noise ratio (SNR)

In arterial phase images, SNR_Liver_ was significantly higher in VMI_40-100keV_ and SNR_HR_ in VMI_40–70keV_ as compared to CI (p<0.05), whereas SNR_AZ_ was higher in VMI_60-200keV_ keV (p<0.05). SNR_RT_ was comparable to CI throughout all keV levels (p>0.05). In portal-venous phase images, VMI_40 – 70 keV_ yielded significantly higher SNR_Liver_ compared to CI (p<0.05), whereas SNR_AZ_ was higher in VMI_70-100keV_ (p<0.05).

#### Contrast to noise ratio (CNR)

CNR_AZ_ was significantly higher in arterial and portal-venous phase VMI_40-70keV_ as compared to CI, respectively (all p<0.05). CNR_HR_ showed significantly higher values in arterial phase VMI_40-60keV_ as compared to CI (all p<0.05). CNR_RT_ was significantly higher than CNR_HR_ in arterial phase images of respective image reconstructions (all p<0.05; Fig 1C).

#### Iodine concentration and iodine perfusion ratio

Iodine concentration and iodine perfusion ratio were significantly higher in RT as compared to HR in arterial phase images (2.7 ± 1.3 vs. 1.6 ± 0.5 mg/ml and 0.2 ± 0.1 vs. 0.1 ± 0.0; all p<0.05; Fig 1D).

### Qualitative analysis

The overall ICC between both readers was 0.925 (0.916–0.932), indicating an excellent agreement.

In CI, AZ shape was rated spherical in 31 and 29 cases (63.3% and 59.2%) and irregular in 18 and 20 cases (36.7% and 40.8%) by reader 1 and 2, respectively. In VMI_50keV_, AZ shape was rated spherical in 34 and 33 cases (69.4% and 67.3%) and irregular in 15 and 16 cases (30.6% and 32.7%) by reader 1 and 2, respectively. HR around the AZ was visible in 39 cases in VMI_50keV_ but only in 24 cases in CI (p<0.05) and appeared discontinuous in 33 (84.6%) and 20 cases (83.3%), respectively.

**Fig 2:**
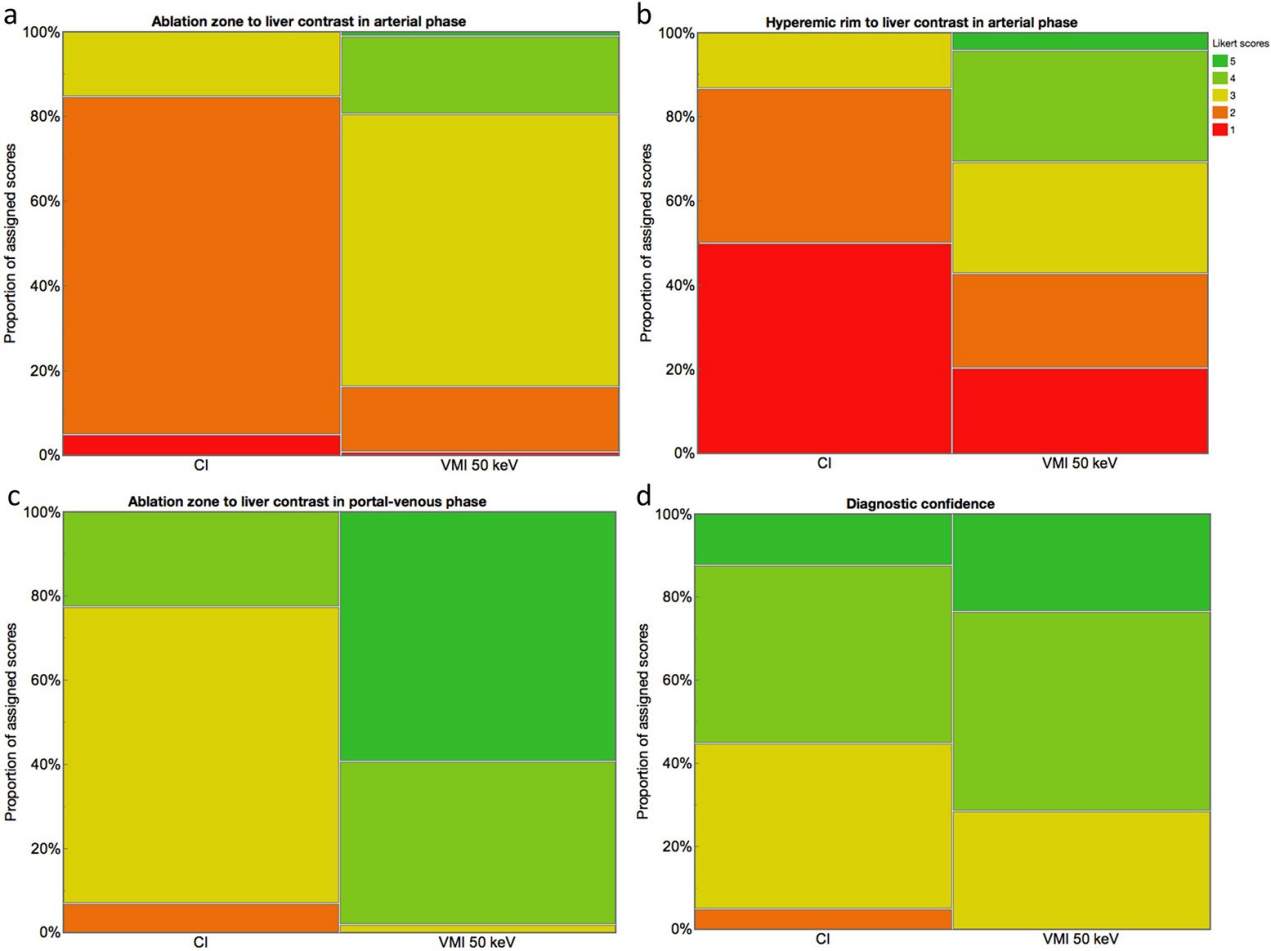
Results of qualitative analysis. Ablation zone as well as hyperemic rim to liver contrast in arterial phase, ablation zone to liver contrast in portal-venous phase and diagnostic confidence received significantly higher ratings in virtual monoenergetic images (VMI) with 50 keV as compared to conventional images (CI) (a–d).

In VMI_50keV_, AZ-to-liver contrast in arterial and portal-venous phase images and HR-to-liver contrast in arterial phase images were rated significantly higher than in CI (all p<0.05, Table 5, Fig 2). Likewise, conspicuity of AZ-margin was rated significantly sharper in VMI_50keV_ as compared to CI (p<0.05). In line with these findings, vessels adjacent to the AZ were better appreciated in VMI_50keV_ in comparison to CI (p<0.05, Table 5).

**Table 5:** Results of qualitative analysis.

	HR-to-liver contrast	AZ-to-liver contrast	Vessel depiction adjacent to AZ	Diagnostic confidence	
Arterial Phase				CI	VMI
CI	1.5 (1–2)	2 (2–2)	3 (2–5)	4 (3–4)	4 (3–4)
VMI	3 (2–4)	3 (3–3)	5 (3–5)		
Portal-venous phase					
CI		3 (3–3)	3 (2–4)		
VMI		5 (4–5)	5 (3–5)		

HR, hyperemic rim; AZ, ablation zone; CI, conventional images; CMI, virtual monoenergetic images. Results are median (interquatile range).

In CI, technique efficacy was categorized as complete in 42 and 39 cases (85.7% and 79.6%), and incomplete in 7 and 10 cases (14.3% and 20.4%) by reader 1 and 2, respectively. In VMI_50keV_, technique efficacy was categorized as complete in 42 and 40 cases (85.7% and 81.6%) and incomplete in 7 and 9 cases (14.3% and 18.4%) by reader 1 and 2, respectively. Assessment of technique efficacy did not significantly differ between reader 1 and 2, nor between CI and VMI_50keV_ (all p≥0.05). Yet, VMI showed a slight benefit over CI for the detection of incomplete ablation with regards to diagnostic accuracy (0.92; sensitivity, 83.3%; specificity, 93.0%) as compared to CI (0.89; sensitivity, 75.0%; specificity, 90.7%). Furthermore, diagnostic confidence was rated significantly higher in VMI_50keV_ compared to CI (p<0.05, Table 5, Fig 2).

## Discussion

This study investigated the value of contrast-enhanced SDCT derived low-keV VMI and iodine maps in the early assessment of technique efficacy after MWA in patients with HCC. It compared CI and VMI derived from early follow-up SDCT after MWA regarding attenuation, image noise, SNR, CNR and it conducted a qualitative analysis of conspicuity of AZ, HR, RT, technique efficacy and diagnostic confidence. Furthermore, the additional value of iodine maps regarding its potential to differentiate reactive hyperemic rim from residual tumor by means of the quantification of iodine concentration and perfusion ratios was assessed.

**Fig 3:**
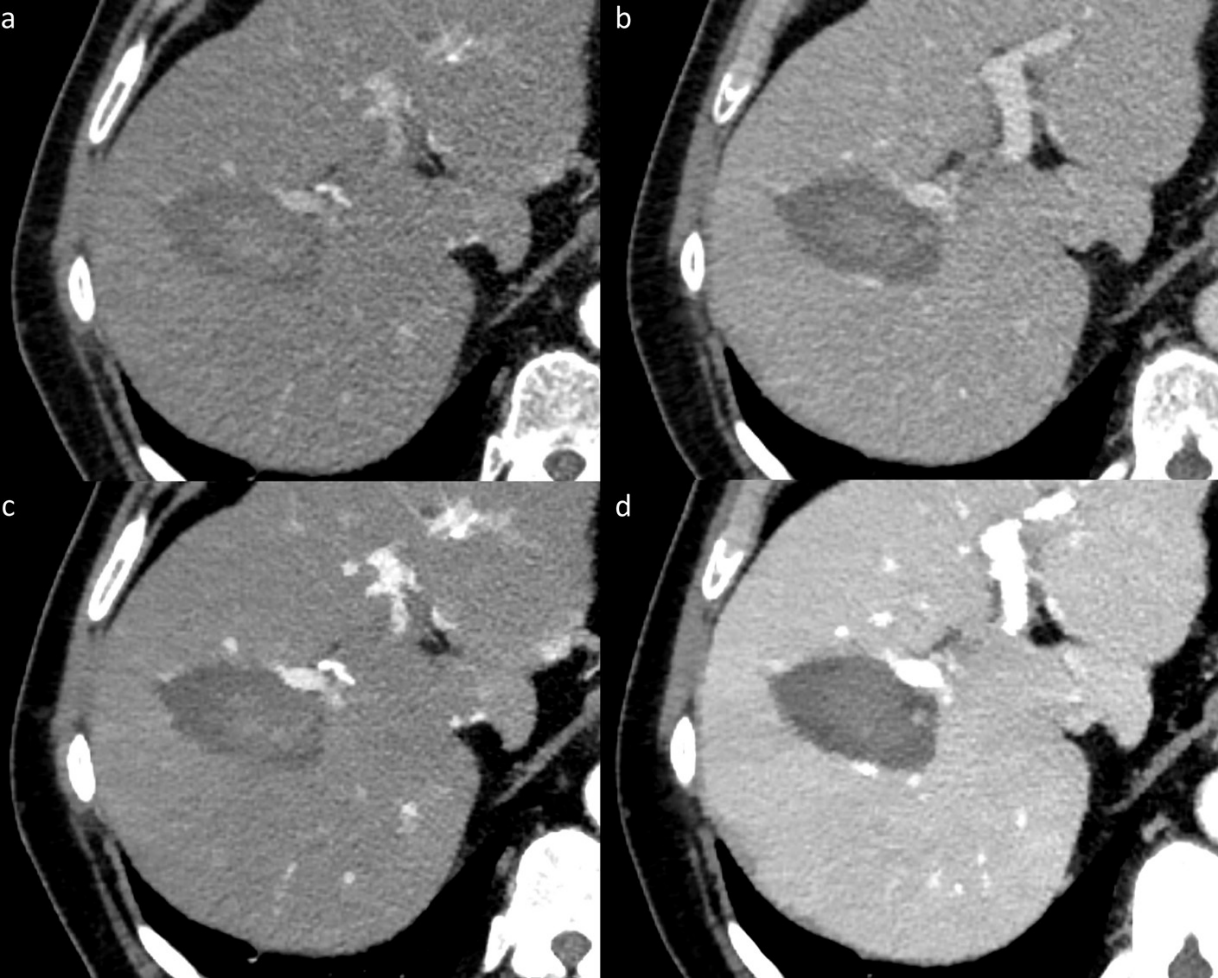
Early follow-up image examples after microwave ablation, illustrating the improved image quality of low-keV virtual monoenergetic images (VMI) (50 keV in c, d) as compared to conventional images (CI) (a, b) in arterial (a, c) and portal-venous phase (b, d). An improved signal-to-noise ratio of the liver parenchyma, contrast-to-noise ratio of the ablation zone, a sharper ablation zone margin and a better depiction of adjacent vessels can be appreciated in the image examples of VMI_50keV_ (c, d) when comparing it with CI (a, b).

**Fig 4:**
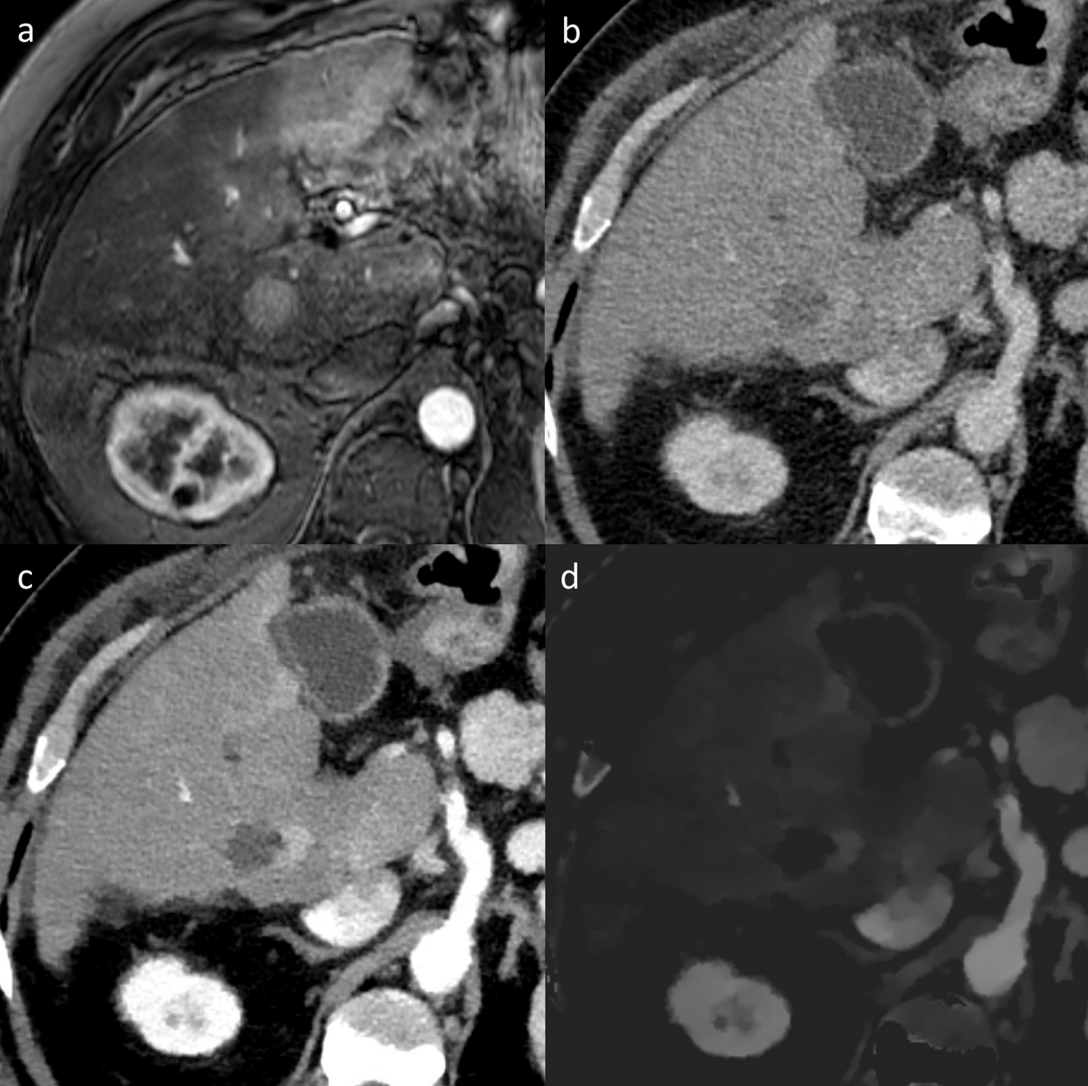
Image examples illustrating the value of spectral detector computed tomography (SDCT) after microwave ablation (MWA) of hepatocellular carcinoma (HCC). The pre-operative magnetic resonance scan clearly shows the arterially hyper-enhancing HCC in liver segment V (a). In the early follow up after incomplete MWA, the detection of residual tumor (RT) is hampered in conventional images (b) due to the poor contrast of the ablation zone and adjacent RT. Using SDCT, RT and thus incomplete ablation can be better depicted by means of low-keV virtual monoenergetic images (50 keV in c) and iodine maps (d).

In low-keV VMI, SNR/CNR of hyperperfused structures such as HR and RT in arterial phase and SNR/CNR of liver parenchyma in arterial and portal-venous phase images were superior to CI due to a boost of iodine attenuation without penalty in image noise [26,28,44]. Low-keV VMI yielded an improved conspicuity of AZ, HR, RT, adjacent vessels and consequently a significant increase of diagnostic confidence (Figs 3 and 4). Furthermore, diagnostic accuracy of technique efficacy was slightly higher in low-keV VMI as compared to CI with an increase in sensitivity and specificity. A comparison with previous studies is limited as to the best of our knowledge, only one clinical study by Lee et al. [34] investigated image quality and diagnostic benefit of DECT derived virtual non-contrast images and iodine maps after thermal liver ablation. The authors reported an improved conspicuity of the AZ after RFA in iodine maps, potentially yielding a better detection of residual tumors [34]. The findings of our study are in line with these previous findings. Two other clinical studies investigated the benefit of DECT for the follow-up after thermal ablation in renal and pulmonary tumors [32,33]. Park et al. evaluated the utility of iodine overlay maps and virtual non-contrast images in the follow-up of renal tumors after RFA and found a comparable diagnostic performance as compared to linear blended CT images with the advantage of a reduction in radiation dose by using virtual non-contrast images [33]. The second study by Izaaryene et al. demonstrated that nodular enhancement, defined as the difference in CT density [HU] between contrast-enhanced and virtual non-contrast images, has the potential to serve as a surrogate for the diff erentiation of scar and local tumor recurrence of lung tumors one month after RFA [32].

Furthermore, we found that attenuation in the respective reconstructions, absolute iodine concentration and normalized iodine perfusion ratio were significantly higher in RT compared to HR. Although the limited number of incomplete ablations in this study made it statistically infeasible to compare the quantitative values regarding their differentiation of HR and RT, these initial results seem promising. To our knowledge, until now, only pre-clinical studies investigated the quantification of iodine as a surrogate parameter in the assessment of treatment response after thermal ablation [29,30]. Zhang et al. reported a better correlation of volumetric iodine concentrations with technical success compared to the modified Response Evaluation Criteria In Solid Tumors (mRECIST) and Choi criteria seven days after hepatic MWA in a rabbit model with VX2 liver carcinoma [29]. Li et al. performed iodine quantification three days, one, two and three weeks after hepatic RFA of VX2 tumors in rabbits to differentiate HR from RT [30]. The authors found that the iodine concentrations differed significantly ≥ two weeks after RFA, but not after three days and one week. Thus, iodine quantification might be employed to differentiate viable RT from HR ≥ two weeks after thermal ablation. A comparison with our retrospective clinical results is limited due to the preclinical design using a rabbit tumor model. Furthermore iodine concentrations not only vary between age groups and genders, but also show intraindividual, longitudinal variation [37,38]. In fact, the reported iodine concentration of residual VX2 tumors differ from our values in RT (one week after thermal ablation 1.7±0.2 mg/ml vs. 2.7±1.3 mg/ml in our study), whereas iodine concentrations of HR appear comparable (one week after thermal ablation 1.6±0.4 mg/ml vs. 1.6±0.5 mg/ml in our study). The results of Li et al. demonstrate that the presence of HR, which is caused by an inflammatory reaction adjacent to the AZ, might obscure RT and therefore hamper early assessment of technique efficacy after thermal ablation [30]. Here, SDCT may be beneficial for an early detection and may facilitate early retreatment of RT.

Despite the single-centre retrospective study design, several limitations need to be addressed. First, the study comprises a limited number of patients and ablated HCC lesions. Second, our results might be affected by our institution´s surveillance schedule. It is known that the AZ size might be underestimated in immediate postinterventional imaging due to tissue contraction induced by thermal ablation [19,45–47]. Yet, there is no specific recommendation by the European, American and Asian hepatology societies with regards to the early assessment of technique efficacy [1,4–6]. While many institutions perform a CT scan after 24 hours and/or one month [14,17,19], others proposed different strategies [15,20,48]. Third, no detailed description of the calculation of SD within the software used for the quantitative analysis is available. Fourth, the number of voxels included in each ROI were not assessed and vary, as ROIs were drawn as large as possible, yet this represents common procedure in radiology research. Fifth, we limited qualitative image analysis to VMI with 50 keV as it represents a previously established low-keV value for the assessment of liver lesions [22,23,25,35]. Furthermore, the determination of threshold values based upon iodine values was out of scope of this study and was statistically infeasible due to the limited number of incomplete ablations. In light of our results, future studies are encouraged to analyze the benefit of iodine values for the early assessment of treatment outcome after MWA and should take the intraindividual variation and recently proposed normalization methods of DECT-derived iodine values into account [38]. Last, we quantitively and qualitatively evaluated the beneficial value of SDCT for the assessment of technique efficacy; however, validation in a larger study and a study with disease-free and overall survival as endpoints are desired.

In conclusion, SDCT derived low-keV virtual monoenergetic images and iodine maps facilitate an improved early assessment of technique efficacy after MWA of HCC compared to CI. The superior image quality in low-keV VMI yielded an improved conspicuity of AZ, HR, RT, adjacent vessels and thus increased diagnostic confidence. Furthermore, absolute iodine values and iodine perfusion ratios may facilitate differentiation of transient inflammatory HR and viable RT.

## Supporting information

S1 Data Complete data. (XLSX)

## Abbreviations

AZ: Ablation zone
CI: Conventional images
CNR: Contrast-to-noise ratio
CT: Computed tomography
DECT: Dual-energy computed tomography
HCC: Hepatocellular carcinoma
HR: Hyperemic rim
HU: Hounsfield units
ICC: Intraclass correlation coefficient
IM: Iodine maps
MRI: Magnetic resonance imaging
MWA: Microwave ablation
RFA: Radiofrequency ablation
ROIs: Regions of interest
RT: Residual tumor
SD: Standard deviation
SDCT: Spectral detector computed tomography
SNR: Signal-to-noise ratio
VMI: Virtual monoenergetic images
